# Molecular Identification of Macroscopic And Microscopic Cysts of Sarcocystis in Sheep in North Khorasan Province, Iran

**Published:** 2014

**Authors:** Pejman Bahari, Mitra Salehi, Mohsen Seyedabadi, Ahmad Mohammadi

**Affiliations:** 1 *Vector-Borne Diseases Research Center, North Khorasan University of Medical & Laboratory of North Khorasan Veterinary Head office,Bojnurd, Iran.*; 2 *Vector-Borne Diseases Research Center, North Khorasan University of Medical, Bojnurd, Iran.*

**Keywords:** *Sarcocystis tenella*, *Sarcocystis gigantea*, *molecular analysis*

## Abstract

Sarcocystis is an obligatory intracellular protozoan parasite which can infect humans and animals. Sheep are intermediate hosts of four *Sarcocystis s*pecies: *Sarcocystis tenella*, *Sarcocystis gigantea*, *Sarcocystis arieticanis,* and *Sarcocystis medusiformis* The purpose of this study was to perform a molecular identification of the macroscopic and microscopic cysts of *Sarcocystis* in sheep. In this investigation, the macroscopic and microscopic cysts of *Sarcocystis* were assessed in slaughtered sheep. The digestion method was used for bradyzoites observation in heart, liver, diaphragm and muscle samples. PCR analysis was conducted on macroscopic and microscopic cysts and also all other samples. Sequencing was performed for ten PCR products. Genotypes were identified by BLAST search and homology analysis. Macrocysts were seen in two muscle tissues. Digestion method and PCR analysis revealed positive results in all samples taken from heart, liver, diaphragm, and muscle. Genotyping of ten tissue samples proved that the genotype of macroscopic belonged to *Sarcocystis gigantea* and microscopic cysts to *Sarcocystis tenella*. Microscopic cysts are more prevalent than macroscopic cysts and they can cause enormous economic losses.

## Introduction

*Sarcocystis* species are intracellular protozoan parasites infecting a wide range of livestocks. Some of *Sarcocystis* genus are pathogenic for animals such as sheep and cattle which cause enormous economic losses (1).

Studies in different regions of the world indicate that the prevalence of *Sarcocystis *infection in slaughtered cattle and sheep are between 70% to 100% (2-6). Additionally, studies in Iran showed that the prevalence of this parasite in the animal was between 85% to 100% (7-13). For example, studies in Kerman and Ahwaz provinces indicated that 100% of animals were infected with *Sarcocystis* (12-13).

Different species of *Sarcocystis *have been isolated from animals worldwide. *Sarcocystis tenella* was isolated from sheep in Iran and Brazil (10, 14). In another study, *Sarcocystis moulei* was reported from reindeer (15). Also, Nourani et al. isolated *Sarcocystis hominis* from cattle (8) while Kalantari et al. separeted* S. cruzi* from cattle (16). Dalimi et al. determined *S. gigantea* and *S. arieticanis* in sheep (9).

Sheep are intermediate hosts of four *Sarcocystis* species: *S. tenella*, *S.gigantea*,* S. arieticanis,* and *S. medusiformis* (1).* S. tenella* and *S. gigantea* have worldwide distribution (1, 9-10, 14). *S. gigantea* and *S. medusiformis* which are non-pathogenic and transferred by cats, can create some macroscopic visible cysts (1). *S. tenella* and *S. arieticanis *are transmitted by dogs and can cause microscopic cysts (1). Pathogenic species in sheep may cause severe disease or abortion during the early phase of infection (1). Sheep will become infected with *S. tenella* by swallowing sporocysts in faces of dog. The first and second meront were created in the asexual stage of *S. tenella* (1). Cysts of *S. tenella* can be found in all sheep tissues. In addition, cysts of *S. tenella *may be formed in the cells of the central nervous system of sheep (1).

In official health control of slaughterhouses, the carcasses infected with macroscopic cysts are removed by veterinarians. But, the microscopic cysts remain because they are not visible. Since, some of microscopic cysts are pathogenic in sheep, thus, we decided to perform the molecular identification of microscopic and macroscopic cysts of this parasite in sheep.

## Materials and Methods

**Sampling method**

This descriptive cross-sectional study was accomplished in eight months, from December 2012 to July 2013. 160 samples were collected from the heart (N= 40), liver (N= 40), diaphragm (N= 40) and muscle (N= 40) from 40 slaughtered sheep (20 males and 20 females) aged between 3 months to 3 years in the slaughterhouses of North Khorasan province.

**Tissue digestion**

Tissue digestion method was used for observing bradyzoite in tissue samples. Seventy grams of each tissue were ground and digested in 1.5% HCL and 0.5% Pepsin at 29°C overnight. The digested samples were filtered through mesh and centrifuged at 1500 rpm for 10 minutes. Then the supernatant was discarded and the pellet was stained by Giemsa and examined microscopically to detect bradyzoites of* Sarcocystis*. For observing bradyzoite in macroscopic cysts; macroscopic cysts were separated from intact tissues and then, both macroscopic cyst and intact tissues were digested, separately.

**DNA extraction and PCR amplification**

For DNA extraction, a small piece of each sample was selected, and DNAs were extracted using tissue DNA extraction kit (Cinnagen company). PCR analysis was performed on all tissue samples using Sar primers including Sar-F1 Forward 5'GCACTTGATGAATTCTGGCA3' and Sar-R1 Reverse 5'CACCACCCATAGAATCAAG 3' (9). PCR reaction was carried out in 30 ml of Ampliqone (Taq DNA polymerase master Mix RED, Denmark). Twenty-five microliters of Taq Master mix were used with 10 ng template DNA, 0.1 μM of each primer and distilled water. Cycles of PCR were set up as follows: Predenaturation step at 94 ° C for 5 min and 30 cycles of denaturation at 94 ° C for 45 s, annealing at 55 °C for 1 min and extension at 72 ° C for 1 min with an elongation step of 7 min at 72 °C at the last cycle. PCR product was electrophoresed on 2% agarose gel, stained with Ethidium Bromide (0.5 μg/ml) and visualized under the UV light.

**Sequencing and genotyping of isolates**

10 PCR products (2 hearts, 2 livers, 2 diaphragms and 4 muscles) were purified. using column-based purification kit and then sequenced through automatic sequencer of the Korean Macrogen Company.

The obtained sequences were edited using Bioedit software program. Genotype identification was performed by comparing with available *Sarcocystis* DNA sequences in the GenBank based on sequence analysis of 18s rRNA region.

## Results

**Fig 1:**
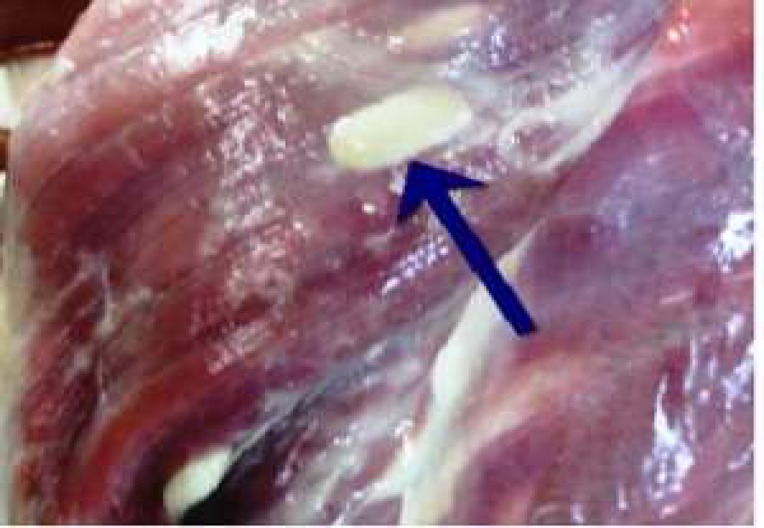
Macroscopic cyst in muscle of sheep

**Fig 2:**
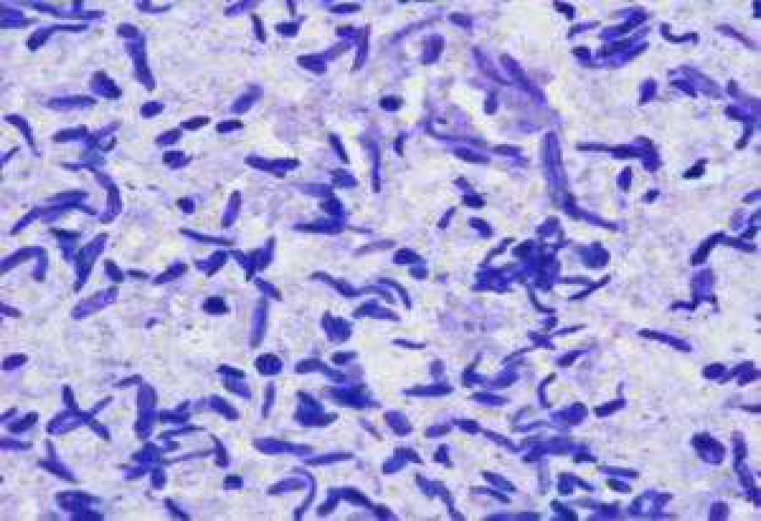
Bradyzoites of Sarcocystis in sheep's muscles

**Fig 3:**
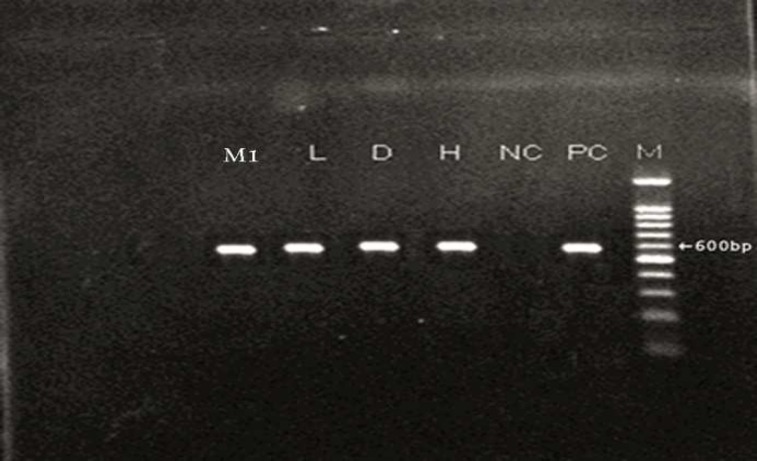
PCR Product from extracted DNA of Sarcocystis in different tissues.

**Fig 4:**
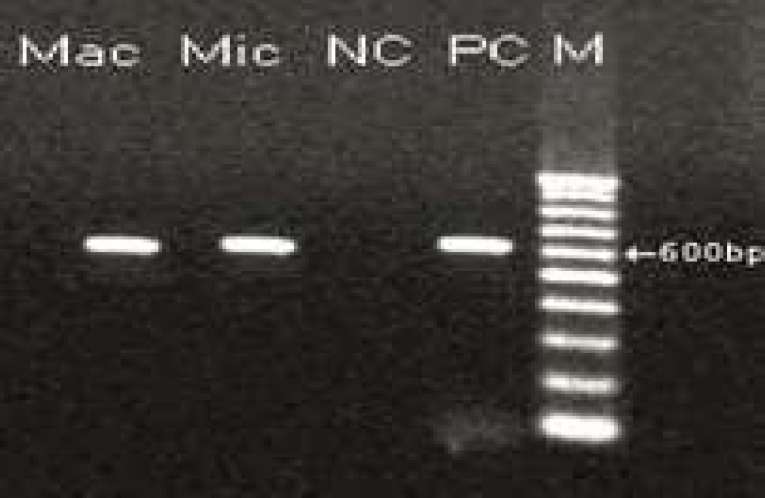
PCR-product of microscopic and macroscopic cyst.

In this study, two samples of muscle had macroscopic cysts (Figure1 and table1). In addition, these muscles were found to be infected with microscopic cysts by digestion method. The results of digestion method showed that all samples of heart, diaphragm, muscle and liver were infected with bradyzoites of *Sarcocystis *(Figure 2). PCR analysis of macrocysts and microcysts as well as all samples showed a specific 600 bp band on the agarose gel (Figures 3 and 4). The results obtained from sequencing of ten samples (2 hearts, 2 diaphragms, 4 muscles and 2 livers) showed that the genotype of macroscopic and microscopic cysts correspond to *Sarcocystis gigantea *and *Sarcocystis tenella,* respectively. 

**Table 1:** Number of macroscopic and microscopic cysts in sheep’s tissues and used methods

	Macroscopic cyst	Microscopic cyst	Digestion method	Molecular method	Negative
Heart	0	40	40	40	0
Diaphragm	0	40	40	40	0
Muscle	2	38	40	40	0
Liver	0	40	40	40	0

The genotypes and accession numbers are shown in table 2.

**Table 2:** Genotypes and accession number of macroscopic and microscopic cyst

ID	Macroscopic cyst	Macroscopic +Microscopic cyst	Accession number	Genotype	
H1	-	-	KF489419	S. tenella	
H2	-	-	KF489427	S. tenella	
D1	-	-	KF489418	S. tenella	
D2	-	-	KF489422	S. tenella	
M1	+	+	KF489421	Mac: S. gigantea	
			KF489428	Mic: S. tenella
M2	2+	2+	KF489425	Mac: S. gigantea	
			KF489429	Mic: S. tenella
L1	-	-	KF489420	S. tenella	
L2	-	-	KF489424	S. tenella	

H: Heart**, **D: Diaphragm**,** M: Muscle, L: Liver**,** Mac: Macroscopic cyst, Mic: Microscopic cyst

M: Molecular weight marker (100bp), PC: Positive Control, NC: Negative Control, H: Heart, D: Diaphragm , L: Liver, M1: Muscle.

## Discussion

This investigation showed that two muscle tissues had macroscopic cysts. Also, all samples (heart, diaphragm, muscle and liver) were infected with *Sarcocystis*spp. The studies in Iran and other parts of the world indicated that livestocks are infected with *Sarcocystis*spp (2-11, 17-19). Other studies in different provinces of Iran showed that 85%-100% of cattle and sheep had *Sarcocystis* infection (7-11, 19).

In previous studies throughout the world, species of *Sacocystis* were isolated from different animals (8-10, 14-15). DaSilva and Langoni isolated *S. tenella* from the sheep in Brazil (14). Al-Hoost et al. reported *S. moulei* from the sheep in Saudi Arabia (20). Moreover, Gjerde isolated and characterized S. grueneri from reindeer based on molecular method (15). In other studies in Iran, *Sarcocystis hominis* and *Sarcocystis cruzi* were studied in cattle (8, 16). Dalimi et al. isolated *S.gigantea* and *S. arieticanis* from the sheep by PCR-RFLP method in Qazvin province, Iran (9). Furthermore, other researchers reported *S. miescheriana* from boar (21) and *S. tenella* from sheep in Iran (10). Additionally, Mahran in Egypt using morphometric method indicated that *S. gigantea* and *S. tenella* caused macroscopic and microscopic cysts (22). Using daub smear method, Bonyadian et al. showed that 91% of cows were infected with microscopic cyst and did not have any macroscopic cysts (23). Kargar Jahromi et al. using digestion method proved that goats had microscopic and macroscopic cysts (24). Molecular analysis was not performed in the above studies, but, the use of molecular methods in the present study showed that *S. gigantea* and *S. tenella *can cause macroscopic and microscopic cysts, respectively. The results of this investigation was in accordance with Anja and Astrid's study who reported that *S. gigantea* and *S. tenella* can cause macroscopic and microscopic cysts, respectively (1).

*S. tenella *is among the pathogenic species and can induce microscopic cysts (1). The severity of clinical symptoms caused by this species depends on the dose of ingested sporocysts and the immune system of the host (1, 25-26).* S. tenella* can lead to acute sarcocystosis in uninfected sheep (1). Non-specific infection symptoms include fever, anorexia, tachycardia and anemia could be observed following infection. In acute sarcocystosis, central nervous system will be involved, and it can cause encephalitis and encephalomyelitis and subsequ-ently death in sheep (27-29). In pregnant sheep, acute sarcocystosis can cause fetal death or premature birth of offspring. Chronic sarcocystosis can create economic problems due to reduced meat, milk and wool (1, 29-30). Also, Dubey reported that *S. tenella* caused symptoms such as inflammation, hepatitis and myocarditis in sheep inoculated with *S. tenella* sporocysts from canine feces (31).

In this study, *S. tenella* was isolated from the heart, diaphragm, muscle and liver of all tested samples. As noted above, *S. tenella* can cause severe clinical signs and dysfunction of organs in sheep, such as tachycardia and neurological disease (1). Furthermore, *Sarcocystis tenella* produces microscopic cyst which are neglected by veterinarians due to its invisible nature. Therefore, improvement of disease control and prevention strategies in sheep would be necessary.

*S.tenella* produces microscopic cyst and which are neglected by veterinarians due to its invisible nature. While *S.gigantea* which causes macroscopic cysts and is non-pathogenic are easily noted. For this reason, macroscopic cysts are removed, but, microscopic cysts do remain. Subsequently, the life cycle of *Sarcocystis tenella* is repeated and it causes enormous economical losses.
